# Pre-Transplant Calcimimetic Use and Dose Information Improves the Accuracy of Prediction of Tertiary Hyperparathyroidism after Kidney Transplantation: A Retrospective Cohort Study

**DOI:** 10.3389/ti.2024.12704

**Published:** 2024-05-01

**Authors:** Manabu Okada, Tetsuhiko Sato, Tomoki Himeno, Yuki Hasegawa, Kenta Futamura, Takahisa Hiramitsu, Toshihiro Ichimori, Norihiko Goto, Shunji Narumi, Yoshihiko Watarai

**Affiliations:** ^1^ Department of Transplant Surgery and Transplant Nephrology, Japanese Red Cross Aichi Medical Center Nagoya Daini Hospital, Nagoya, Aichi, Japan; ^2^ Department of Diabetes and Endocrinology, Japanese Red Cross Aichi Medical Center Nagoya Daini Hospital, Nagoya, Aichi, Japan

**Keywords:** calcimimetics, kidney transplantation, parathyroidectomy, tertiary hyperparathyroidism, prediction model

## Abstract

Tertiary hyperparathyroidism (THPT) is characterized by elevated parathyroid hormone and serum calcium levels after kidney transplantation (KTx). To ascertain whether pre-transplant calcimimetic use and dose information would improve THPT prediction accuracy, this retrospective cohort study evaluated patients who underwent KTx between 2010 and 2022. The primary outcome was the development of clinically relevant THPT. Logistic regression analysis was used to evaluate pre-transplant calcimimetic use as a determinant of THPT development. Participants were categorized into four groups according to calcimimetic dose, developing two THPT prediction models (with or without calcimimetic information). Continuous net reclassification improvement (CNRI) and integrated discrimination improvement (IDI) were calculated to assess ability to reclassify the degree of THPT risk by adding pre-transplant calcimimetic information. Of the 554 patients, 87 (15.7%) developed THPT, whereas 139 (25.1%) received pre-transplant calcimimetic treatment. Multivariate logistic regression analysis revealed that pre-transplant calcimimetic use was significantly associated with THPT development. Pre-transplant calcimimetic information significantly improved the predicted probability accuracy of THPT (CNRI and IDI were 0.91 [*p* < 0.001], and 0.09 [*p* < 0.001], respectively). The THPT prediction model including pre-transplant calcimimetic information as a predictive factor can contribute to the prevention and early treatment of THPT in the era of calcimimetics.

## Graphical Abstract

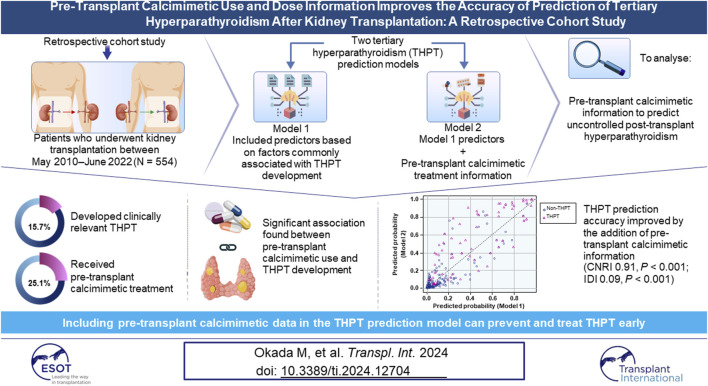

## Introduction

Persistent hyperparathyroidism after kidney transplantation (KTx) is associated with unfavorable kidney graft and patient outcomes [1–3]. Tertiary hyperparathyroidism (THPT) is characterized by high parathyroid hormone (PTH) and serum calcium (Ca) levels, even in functioning kidney grafts [4], and often requires therapeutic intervention [5–8]. Common treatment options for THPT include parathyroidectomy (PTx) and calcimimetics [9–11]. However, in KTx patients, PTx can increase serum creatinine levels [12, 13], and the disadvantages of calcimimetics include being off-label in some regions, high medical costs [14], and an increased risk of urinary stones [15, 16]. For patients at high risk of THPT, pre-transplant PTx is appropriate [17, 18].

The predictive factors for THPT include pre-transplant serum Ca and PTH levels, dialysis duration, and parathyroid gland size [19, 20]. Prediction models using only three variables (serum Ca, PTH levels, and dialysis duration) have been shown to accurately predict the risk of THPT [21]. However, recently, pre-transplant calcimimetic administration has also been reported as an additional predictive factor for THPT [22, 23].

The effectiveness of calcimimetics in the treatment of secondary hyperparathyroidism (SHPT) is widely recognized. In vitamin D-resistant SHPT, cinacalcet effectively reduces PTH levels [24, 25]. Several studies have demonstrated that cinacalcet prevents cardiovascular events and patient mortality [26–28]. Following cinacalcet, new calcimimetics have been developed [29, 30], and with an increase in treatment options, the proportion of dialysis patients receiving calcimimetic treatment is likely to increase. In this era of calcimimetics, pre-transplant calcimimetic use and dose information may predict THPT progression after KTx.

THPT risk assessment is complicated by several factors. In patients treated with calcimimetics, the assessment of THPT risk can be challenging because of the drastic decrease in serum Ca and PTH levels [31, 32]. Cianciolo et al. [33] proposed evaluating the need for PTx in KTx candidates receiving calcimimetic treatment after ceasing treatment for 2–4 weeks. However, discontinuation of calcimimetic treatment leads to a rapid increase in PTH levels, which may cause hyperparathyroidism-related adverse events and complicate the optimal timing of KTx. Therefore, assessment of THPT risk without discontinuing calcimimetic treatment is safer. A need for highly accurate prediction of THPT risk arises; this can contribute to the prevention and early treatment of THPT in patients undergoing KTx. Accurate THPT prediction models that include calcimimetic dose information are therefore required.

Hence, in this retrospective study, we aimed to investigate whether the inclusion of calcimimetic use and dose information as predictive factors in a prediction model could improve THPT prediction accuracy.

## Materials and Methods

### Data Source

Consecutive patients who underwent KTx between May 2010 and June 2022 were included. The data were collected on 30 June 2023.

### Participants

The exclusion criteria were as follows: 1) PTx before KTx, 2) end-stage kidney disease with an estimated glomerular filtration rate (eGFR) of less than 15 mL/min/1.73 m^2^ within a year after KTx, 3) denosumab treatment within a year after KTx, 4) missing data, and 5) preemptive KTx. Data on patient age, sex, body mass index, original disease, dialysis duration, serum Ca and intact PTH levels, kidney graft function, parathyroid gland size (the size of the parathyroid glands of recipients were routinely measured by ultrasound before KTx), ABO blood type incompatibility, positivity for donor-specific human leukocyte antigen antibodies, and PTx and calcimimetic treatment histories, were collected.

All procedures involving participants were approved by the Institutional Review Board (IRB) and performed in accordance with the 1964 Helsinki Declaration and its later amendments or comparable ethical standards. The IRB waived the requirement to obtain informed consent because of the retrospective nature of the study. Details of the study and its outcomes are available on our institutional website. This study was conducted in accordance with the Strengthening the Reporting of Observational Studies in Epidemiology guidelines.

### Outcome

The primary outcome was the development of clinically relevant THPT, defined as the presence of both hypercalcemia (total serum Ca ≥10.5 mg/dL) and high PTH level (intact PTH >80 pg/mL) 1 year after KTx, based on the guidelines of the Japanese Society for Dialysis Therapy [6, 34]. In addition, post-transplant PTx or calcimimetic therapy to control severe hyperparathyroidism was included in the definition of THPT.

### Measurements

Pre-transplant blood sample analyses were performed in all patients within 3 months before KTx. Serum Ca levels were measured using standard methods. Intact PTH levels were measured using the following second-generation immunoassays: an electrochemical luminescence immunoassay (SRL, Tokyo, Japan^1^, reference range 10–65 pg/mL) and an enzyme immunoassay (Tosoh, Tokyo, Japan^2^, reference range 9–80 pg/mL). For serum albumin levels <4.0 g/dL, all serum Ca levels were corrected [35]. The eGFR was evaluated using the creatinine equation provided by the Japanese Society of Nephrology and the Japanese Society for Pediatric Nephrology [36, 37].

### Immunosuppression

Immunosuppressive regimens included calcineurin inhibitors (cyclosporine or tacrolimus), mycophenolic acids, mizoribine, everolimus, and glucocorticoids. Basiliximab was used as induction therapy. In addition, rituximab administration or splenectomy was used as induction therapy in anti-donor antibody-positive patients before KTx, except in those with low antibody titers.

### Statistical Analysis

Pearson’s chi-squared test was used to analyze nominal variables, and the Mann–Whitney U test or Student’s *t*-test was used for continuous variables. The normality of the distribution of the data was assessed using the Shapiro–Wilk normality test and histogram (Supplementary Table S1; Supplementary Figure S1). Statistical significance was set at *p* < 0.05.

First, logistic regression analysis was performed to confirm that known predictive factors were associated with the development of THPT, even after adjusting for the patient background between the THPT and non-THPT groups. Then, two THPT prediction models were constructed using logistic regression, one with and one without pretransplant calcimimetic use and dose information (Model 1 and Model 2). Owing to the non-linear relationship between serum Ca, intact PTH, dialysis duration, parathyroid gland size, and THPT risk (Supplementary Figure S1), these variables were transformed into categorical variables by dividing them into four categories based on the number of cases. The information on pre-transplant calcimimetic treatment was also used to categorize participants into four groups according to the tertile of cinacalcet dose per unit of body weight (mg/kg). Based on previous studies, evocalcet (2.0 mg/day) and etelcalcetide (7.5 mg/week) dosages were considered equivalent to a cinacalcet dosage of 25.0 mg/day [38, 39].

To evaluate the effect of the inclusion of pre-transplant calcimimetic information as a predictive factor for THPT, the accuracy of Models 1 and 2 were compared. First, scatter plots of the predicted probabilities of Models 1 and 2 were created, then continuous net reclassification improvement (CNRI) and integrated discrimination improvement (IDI) were calculated to assess the ability to reclassify the degree of THPT risk by adding pretransplant calcimimetic information [40–42]. To identify the characteristics of THPT patients for whom the addition of the pre-transplant calcimimetic information significantly improved the predictive probability, we stratified THPT cases by a change in predictive probability of 0.1 and compared the characteristics. In addition, receiver operating characteristic (ROC) curves for the predicted THPT probabilities of each model were obtained, and the areas under the curve (AUCs) were compared for the two models using Delong’s test [43].

### Internal Validation

Internal validation of the prediction models was performed using the bootstrap method [44]. By resampling with replacement, 1,000 pseudo-external datasets were created, and the ROC AUC was obtained. Overfitting was assessed using slope optimism, and calibration was performed.

Easy R (EZR) version 1.61 (The R Foundation for Statistical Computing) was used for the statistical analyses [45]. The calculations of CNRI and IDI, as well as the internal validation by the bootstrap method, were performed using the R package “rms” (version 6.7–0). Statistical significance was set at *p* < 0.05.

## Results

### Participant Characteristics

**FIGURE 1:**
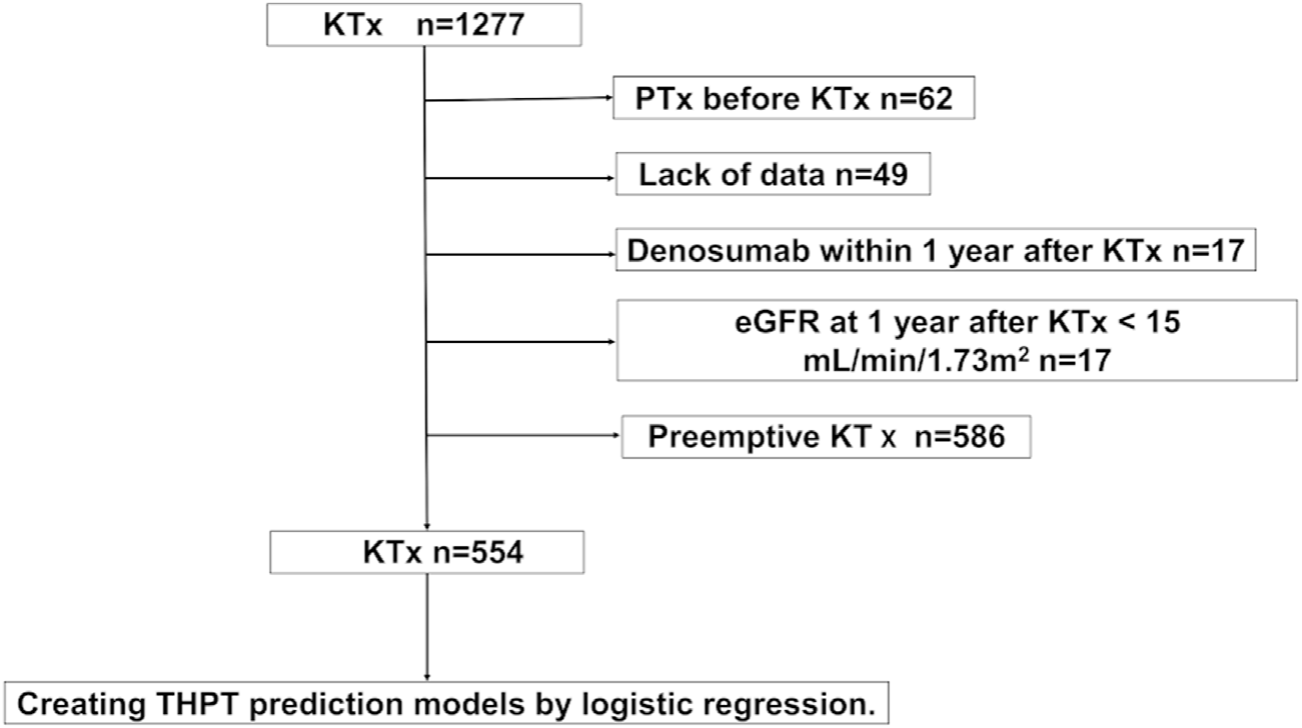
Participant selection flowchart. eGFR, estimated glomerular filtration rate; KTx, kidney transplantation; PTx, parathyroidectomy; THPT, tertiary hyperparathyroidism.

A total of 554 patients met the inclusion criteria (median observation period, 81 months [interquartile range {IQR}: 47–122 months]; Figure 1). Of the 554 patients, 87 (15.7%) developed THPT after KTx, whereas 139 (25.1%) received calcimimetic treatment before KTx (Table 1, Supplementary Table S2). More than 70% of patients had pre-transplant hyperparathyroidism (i-PTH >80 pg/mL) with or without pre-transplant calcimimetic treatment (Supplementary Table S3). Significant differences were observed between the THPT and non-THPT groups in terms of dialysis duration, living donor, parathyroid gland size, pre-transplant calcimimetic use, and serum Ca and intact PTH levels (Table 1). In addition, serum Ca and intact PTH levels 1 year after KTx also significantly differed between the two groups (Table 2). In the THPT group (*n* = 87), 43 (49.4%) received PTx, and 36 (41.4%) received calcimimetic treatment after KTx (Table 2). Most PTx were done within 2 years after KTx (the median interval from KTx to PTx was 10.0 months [IQR: 7–17 months]), and post-transplant calcimimetic treatment was initiated within 1 year after KTx in all cases (Table 2).

**TABLE 1:** Patient characteristics before KTx.

	Total N = 554	Non-THPT N = 467	THPT N = 87	*p*-value
Recipient age (years, IQR)	51 (39–62)	50 (38–62)	53 (46–62)	0.060
Recipient sex (male, %)	352 (63.5)	304 (65.1)	48 (55.2)	0.089
Body mass index (kg/m^2^, SD)	22.1 (3.7)	22.1 (3.8)	22.0 (3.3)	0.807
Dialysis vintage (months, IQR)	21 (6–54)	16 (5–38)	112 (48–167)	<0.001*
Previous KTx (%)	22 (4.0)	18 (3.9)	4 (4.6)	0.764
Living donor (%)	506 (91.3)	438 (93.8)	68 (78.2)	<0.001*
Original disease (%)				0.058
Glomerular disease	192 (34.7)	159 (34.0)	33 (37.9)	
Diabetic kidney disease	141 (25.6)	122 (26.1)	19 (21.8)	
Polycystic kidney disease	28 (5.1)	19 (4.1)	9 (10.3)	
Hypertensive kidney disease	38 (6.9)	36 (7.7)	2 (2.3)	
Others	49 (8.8)	39 (8.4)	10 (11.5)	
Unknown	106 (19.1)	92 (19.7)	14 (16.1)	
Preformed DSA (%)	40 (7.2)	38 (8.1)	2 (2.3)	0.068
ABO blood type incompatible kidney transplantation (%)	160 (28.9)	128 (27.4)	32 (36.8)	0.093
Parathyroid gland size (mm, IQR)	7.2 (5.1–9.8)	6.3 (4.7–8.4)	9.4 (7.1–11.6)	<0.001*
VDRA before KTx (%)	352 (63.5)	288 (61.7)	64 (73.5)	0.039*
Alfacalcidol	184 (33.2)	164 (35.1)	20 (23.0)	
Calcitriol	64 (11.5)	47 (10.1)	17 (19.5)	
Maxacalcitol	104 (18.8)	77 (16.5)	27 (31.0)	
Calcimimetics before KTx (%)	139 (25.1)	84 (18.0)	55 (63.2)	<0.001*
Cinacalcet	89 (16.1)	50 (10.7)	39 (44.8)	
Evocalcet	36 (6.5)	25 (5.4)	11 (12.6)	
Etelcalcetide	14 (2.5)	9 (1.9)	5 (2.7)	
Calcimimetic dose per unit of body weight (mg/kg, IQR)	0.4 (0.3–0.7)	0.4 (0.3–0.5)	0.6 (0.4–1.0)	<0.001*
Lab data before KTx				
Corrected calcium (mg/dL, IQR)	9.3 (8.9–9.8)	9.2 (8.9–9.7)	9.8 (9.3–10.3)	<0.001*
Intact PTH (pg/mL, IQR)	157.5 (85.0–248.0)	145.0 (78.0–240.0)	203 (154.5–317.5)	<0.001*

*DSA*, donor-specific HLA antibody; *eGFR*, estimated glomerular filtration rate; *IQR*, interquartile range; *KTx*, kidney transplantation; *PTH*, parathyroid hormone; *SD*, standard deviation; *THPT*, tertiary hyperparathyroidism; *VDRA*, vitamin D receptor activator.

The results of parathyroid gland size excluded patients in whom parathyroid gland was not detected by echography.

Calcimimetic dose was converted into cinacalcet dose and calculated by per unit of body weight, excluding patients who had not received pre-KTx calcimimetic treatment.

*p-value <0.05.

**TABLE 2:** Clinical data after KTx.

	Total N = 554	Non-THPT N = 467	THPT N = 87	*p*-value
Lab data 1 year post-KTx				
Corrected calcium (mg/dL, IQR)	9.7 (9.4–10.0)	9.7 (9.4–9.9)	10.6 (9.8–10.8)	<0.001*
Intact PTH (pg/mL, IQR)	91.0 (65.0–130.0)	86.0 (64.2–115.0)	137.0 (88.9–181.0)	<0.001*
Recipient eGFR (mL/min/1.73 m^2^, IQR)	44.2 (36.9–51.8)	43.1 (36.4–51.2)	44.2 (36.5–52.1)	0.695
Parathyroidectomy after KTx (%)	43 (4.0)	0 (0.0)	43 (49.4)	<0.001*
Interval between KTx and PTx				NA
<=12 months	NA	NA	25 (58.1%)	
13–24 months	NA	NA	14 (32.6)	
>24 months	NA	NA	4 (9.3)	
Calcimimetics after KTx (%)	36 (3.1)	0 (0.0)	36 (41.4)	<0.001*
Follow up after KTx (months, IQR)	81 (47–122)	81 (47–122)	89 (55–119)	0.371

*eGFR*, estimated glomerular filtration rate; *IQR*, interquartile range; *KTx*, kidney transplantation; *NA*, not applicable; *PTH*, parathyroid hormone; *PTx*, parathyroidectomy; *THPT*, tertiary hyperparathyroidism.

*p-value <0.05.

### THPT Predictive Factors

Multivariate logistic regression analysis of predictive factors for THPT development revealed that dialysis duration, pre-transplant serum Ca levels, intact PTH levels, parathyroid gland size, and pre-transplant calcimimetic use were significantly associated with THPT (Table 3).

**TABLE 3:** Logistic regression for THPT development.

	Univariate			Multivariate		
Factors	OR	95% CI	*p-*value	OR	95% CI	*p*-value
Living donor	0.24	0.13–0.45	<0.001*	0.73	0.25–2.14	0.568
Preformed DSA	0.27	0.06–1.12	0.071	0.12	0.01–1.48	0.098
Pretransplant VDRA use	1.73	1.04–2.88	0.036*	1.90	0.87–4.16	0.109
Dialysis duration (months, reference to <6)						
6–20	0.75	0.24–2.28	0.609	0.88	0.24–3.22	0.841
21–53	1.50	0.56–3.99	0.419	0.62	0.18–2.18	0.457
54–	14.30	6.21–32.70	<0.001*	6.99	2.26–21.70	<0.001*
Serum Ca before KTx (mg/dL, reference to <8.9)						
8.9–9.2	0.76	0.29–2.00	0.581	1.39	0.37–5.21	0.627
9.3–9.7	2.67	1.23–5.77	0.013*	4.58	1.51–13.90	0.007*
9.8–	5.35	2.56–11.20	<0.001*	16.90	5.16–55.20	<0.001*
Intact PTH before KTx (pg/mL, reference to <85.0)						
85.0–157.0	3.27	1.26–8.52	0.015*	11.50	2.96–44.70	<0.001*
158.0–247.0	6.29	2.52–15.70	<0.001*	19.30	5.38–69.30	<0.001*
248.0–	6.66	2.69–16.50	<0.001*	28.50	7.65–106.00	<0.001*
Parathyroid gland size before KTx (mm, reference to 0)						
0.1–5.7	2.10	0.90–4.86	0.085	1.34	0.45–3.99	0.602
5.8–8.8	4.79	2.40–9.57	<0.001*	3.53	1.32–9.44	0.012*
8.9–	17.60	9.27–33.40	<0.001*	12.30	4.46–34.00	<0.001*
Pretransplant calcimimetics use	7.84	4.77–12.90	<0.001*	10.80	4.73–24.60	<0.001*

*Ca*, Calcium; *95% CI*, 95% confidence interval; *DSA*, donor-specific HLA antibody; *KTx*, kidney transplantation; *OR*, odds ratio; *PTH*, parathyroid hormone; *THPT*, tertiary hyperparathyroidism; *VDRA*, vitamin D receptor activator.

The parathyroid gland size was defined as 0 when parathyroid gland was not detected by echography.

*p-value <0.05.

### THPT Prediction Models

Two THPT prediction models were created based on the logistic regression analysis. Model 1 was created from four predictors: dialysis duration, serum Ca level, intact PTH level, and parathyroid gland size, whereas Model 2 was created by adding the calcimimetic dose per unit of body weight to the predictors used in Model 1 (Table 4).

**TABLE 4:** Logistic regression THPT prediction models.

	Model 1			Model 2		
Variable	RC (SE)	OR (95% CI)	*p*-value	RC (SE)	OR (95% CI)	*p*-value
(Intercept)	−6.26 (0.79)			−7.57 (0.94)		
Dialysis duration (months, reference to < 6)						
6–20	−0.07 (0.62)	0.94 (0.28–3.13)	0.913	−0.19 (0.67)	0.83 (0.87–3.05)	0.775
21–53	0.11 (0.57)	1.11 (0.36–3.41)	0.852	−0.52 (0.65)	0.59 (0.17–2.13)	0.423
54–	2.40 (0.50)	11.0 (4.12–29.60)	<0.001	1.84 (0.56)	6.27 (2.10–18.70)	0.001
Serum Ca (mg/dL, reference to < 8.9)						
8.9–9.2	−0.42 (0.58)	0.66 (0.21–2.06)	0.470	0.23 (0.68)	1.26 (0.33–4.80)	0.736
9.3–9.7	1.07 (0.57)	2.91 (1.11–7.58)	0.029	1.43 (0.56)	4.18 (1.38–12.60)	0.011
9.8–	1.82 (0.50)	6.20 (2.33–16.50)	<0.001	2.70 (0.59)	15.00 (4.72–47.40)	<0.001
Intact PTH (pg/mL, reference to < 85.0)						
85.0–157.0	1.55 (0.58)	4.71 (1.51–14.70)	0.008	2.27 (0.66)	9.69 (2.65–35.40)	0.001
158.0–247.0	2.70 (0.58)	14.90 (4.80–46.50)	<0.001	2.85 (0.63)	17.40 (5.00–60.20)	<0.001
248.0–	2.63 (0.58)	13.8 (4.44–43.20)	<0.001	3.17 (0.64)	23.80 (6.73–83.90)	<0.001
Parathyroid gland size (mm, reference to 0)						
0.1–5.7	0.83 (0.50)	2.29 (0.86–6.08)	0.096	0.30 (0.55)	1.35 (0.46–3.97)	0.579
5.8–8.8	1.45 (0.46)	4.27 (1.74–10.50)	0.002	1.28 (0.49)	3.61 (1.37–9.50)	0.009
8.9–	2.54 (0.44)	12.60 (5.31–30.00)	<0.001	2.33 (0.53)	10.20 (3.65–28.80)	<0.001
Calcimimetic dose per unit of body weight (mg/kg, reference to 0)						
0.1–0.2	NA	NA	NA	1.88 (0.60)	6.54 (2.04–21.00)	0.002
0.3–0.4	NA	NA	NA	2.23 (0.58)	9.32 (3.02–28.80)	<0.001
0.5–	NA	NA	NA	2.95 (0.55)	19.10 (6.55–55.70)	<0.001

*Ca*, calcium; *95% CI*, 95% confidence interval; NA, not applicable; *OR*, odds ratio; *PTH*, parathyroid hormone; *RC*, regression coefficient; *SE*, standard error.

The parathyroid gland size was defined as 0 when parathyroid gland was not detected by echography.

Calcimimetic dose was converted into cinacalcet dose and calculated by per unit of body weight and is only adopted as a predictive factor in Model 2.

### Effect of the Pre-Transplant Calcimimetic Information on THPT Prediction

**FIGURE 2:**
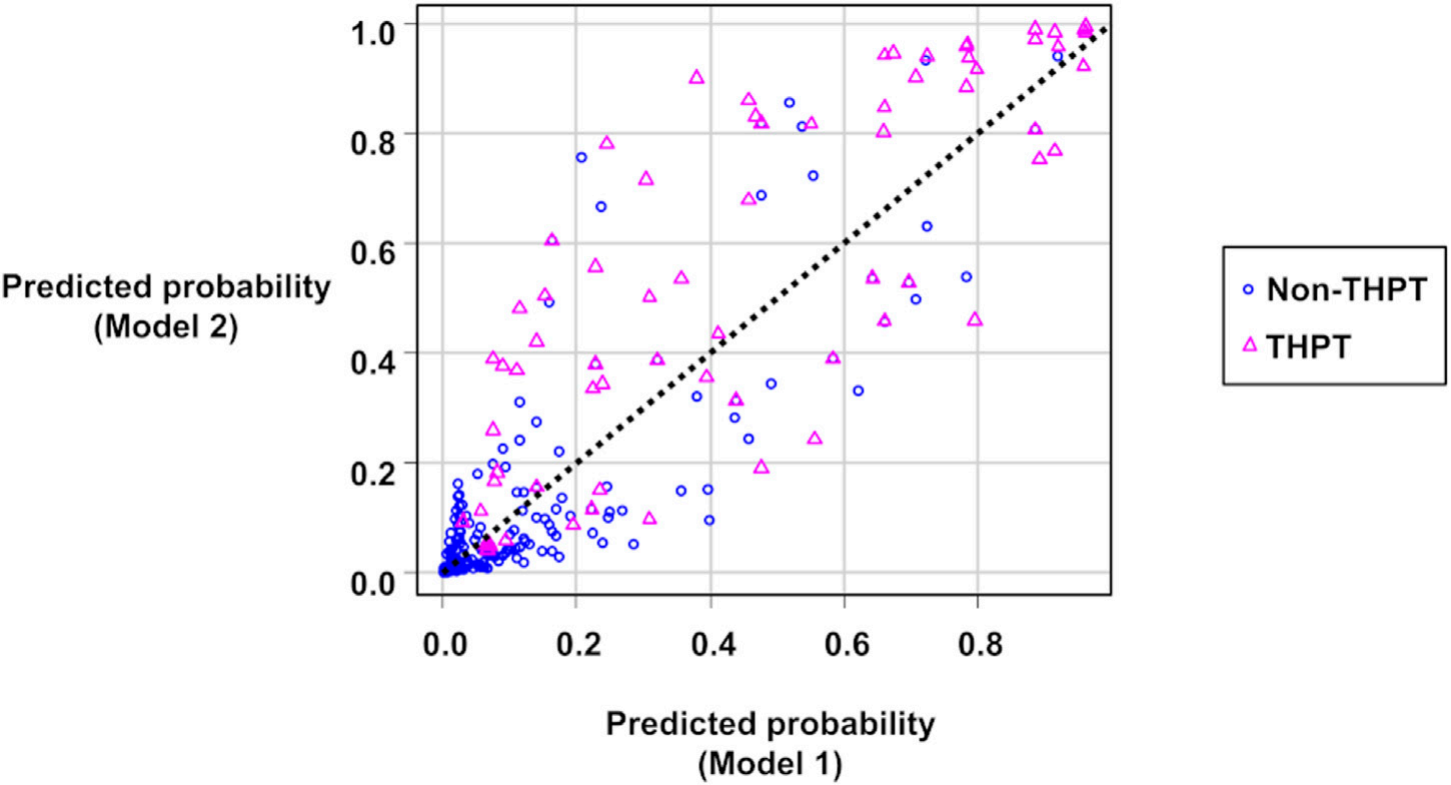
Scatter plots of the predicted probabilities of Model 1 and Model 2. The circles represent non-THPT cases, and the triangles represent THPT cases. The black dashed line represents the coordinates where the predictions of Model 1 and Model 2 match. The circles below the black dashed line or the triangles above it indicate that the THPT predictions have improved in Model 2 compared with Model 1. THPT, tertiary hyperparathyroidism.

Figure 2 shows scatter plots of the predicted probabilities of Models 1 and 2. When comparing the predicted probabilities of the two THPT prediction models, the addition of the pre-transplant calcimimetic information improved the predicted probabilities in 65.5% (57/87) of the THPT group and 80.1% (374/467) of the non-THPT group, respectively (Figure 2; Table 5). The CNRI calculated from the sum of the proportion of improvement/worsening of the predicted probabilities was 0.91 (95% CI: 0.70–1.13, *p* < 0.001) (Figure 2; Table 5). In contrast, the mean changes in predicted probabilities were 0.08 in the THPT group and 0.01 in the non-THPT group, resulting in an IDI of 0.09 (95% CI: 0.05–0.13, *p* < 0.001) (Figure 2; Table 5). In the subgroup of THPT with an improvement of 0.1 or more in predictive probabilities by adding the pre-transplant calcimimetic information, both the proportion of patients receiving pretransplant calcimimetics and the doses of pre-transplant calcimimetics were significantly higher (Table 6).

**TABLE 5:** Summary of the calculation for CNRI and IDI for Model 2 compared to Model 1.

Proportions of positive and negative changes in predicted probabilities				
(1) Increase of predicted probability for THPT group: 0.655 (57/87)				
(2) Increase of predicted probability for non-THPT group: 0.199 (93/467)				
(3) Decrease of predicted probability for THPT group: 0.345 (30/87)				
(4) Decrease of predicted probability for non-THPT group: 0.801 (374/467)				
**CNRI**	**Index (SE)**	**Z value**	***p*-value**	**95% CI**
CNRI for THPT group (1–3)	0.31 (0.10)	3.05	0.002*	0.11–0.51
CNRI for non-THPT group (4–2)	0.60 (0.04)	16.28	<0.001*	0.53–0.67
CNRI for entire cohort (1–3+4–2)	**0.91 (0.11)**	8.4	< 0.001*	0.70–1.13
**Mean change in predicted probability**				
Increase for THPT group (sensitivity): 0.08				
Decrease for non-THPT group (specificity): 0.01				
**IDI**	**Index (SE)**	**Z value**	***p*-value**	**95% CI**
		**0.09 (0.02)**	4.35	<0.001*	0.05–0.13

*95% CI*, 95% confidential interval; *CNRI*, continuous net reclassification improvement; *IDI*, integrated discrimination improvement; *THPT*, tertiary hyperparathyroidism; *SE*, standard error.

*p-value <0.05.

The bold values represent the final results of the analysis.

**TABLE 6:** Characteristics of THPT patients classified by degree of improvement in predicted probability.

	PP improvement <0.1 *n* = 48	PP improvement >=0.1 *n* = 39	*p*-value
Dialysis duration (months, IQR)	95 (45–146)	123 (67–171)	0.294
Serum Ca before KTx (mg/dL, IQR)	9.9 (9.50–10.4)	9.6 (9.0–10.0)	0.059
Serum intact PTH before KTx (pg/mL, IQR)	239.5 (177.3–341.8)	190.0 (122.0–286.5)	0.067
Parathyroid gland size (mm, IQR)	9.0 (0.0–11.0)	5.5 (0.0–8.80)	0.05
Pre-transplant calcimimetic treatment (%)	16 (33.3)	39 (100.0)	<0.001*
Pre-transplant calcimimetic dose per unit of body weight (mg/kg, IQR)	0.0 (0.0–0.3)	0.7 (0.4–1.1)	<0.001*

*Ca*, calcium; *IQR*, interquartile range; *KTx*, kidney transplantation; *PTH*, parathyroid hormone; *PP*, predicted probability; *THPT*, tertiary hyperparathyroidism.

Calcimimetic dose was converted into cinacalcet dose and calculated by per unit of body weight.

*p-value <0.05.

**FIGURE 3:**
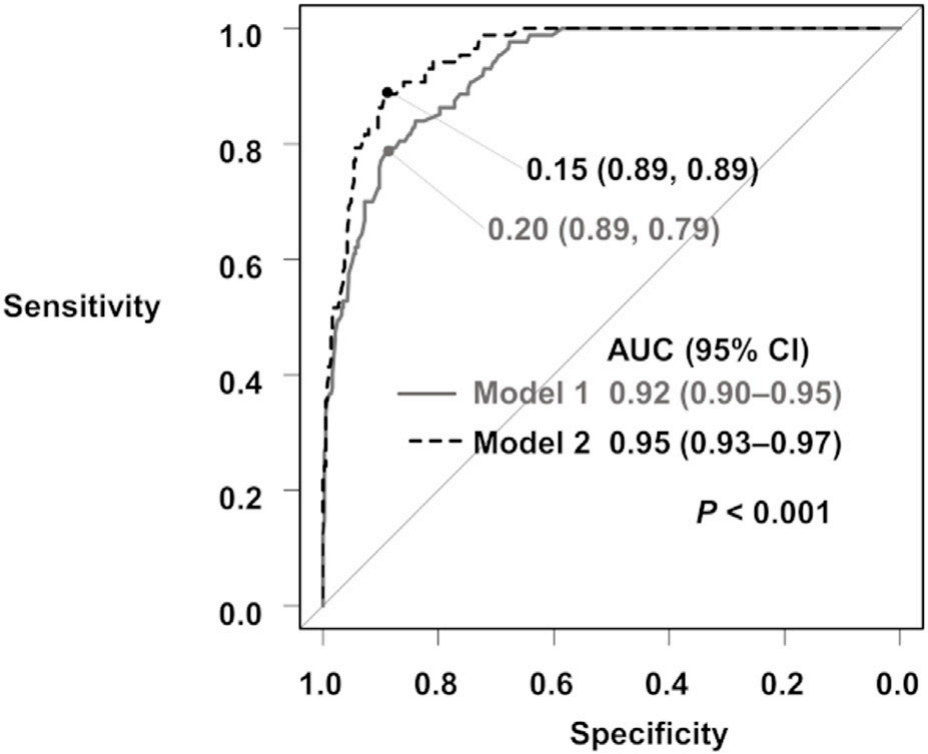
ROC curves for the prediction of THPT from Model 1 and Model 2. The gray curve is the ROC curve for Model 1, and the black dashed curve is the ROC curve for Model 2. The ROC AUCs and 95% CIs are shown. AUC, area under the curve; 95% CI, 95% confidence interval; ROC, receiver operating characteristic; THPT, tertiary hyperparathyroidism.

When comparing the ROC AUCs of the two THPT prediction models, the inclusion of the pretransplant calcimimetic information significantly improved the AUC from 0.92 (95% CI: 0.90–0.95, cut-off value: 0.20, specificity: 0.89, sensitivity: 0.79) to 0.95 (95% CI: 0.93–0.97, cut off value: 0.15, specificity: 0.89, sensitivity: 0.89) (*p* < 0.001) (Figure 3; Supplementary Table S4).

### Internal Validation of THPT Prediction Models

**FIGURE 4:**
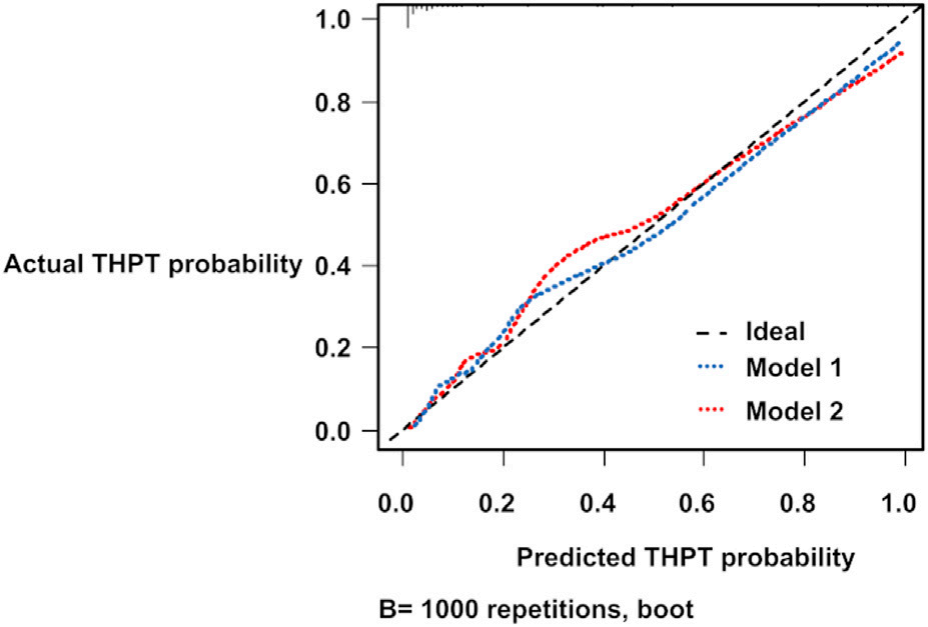
Calibration diagrams for THPT prediction models using the bootstrap method. The blue and red dashed lines represent the calibration diagrams for Model 1 and Model 2, respectively. THPT, tertiary hyperparathyroidism.

The bootstrapped ROC AUCs for Models 1 and 2 were 0.91 and 0.94, respectively (Table 7). The slope optimism values of the two models were 0.11 and 0.16, respectively (Table 7). From the calibration diagrams based on the bootstrap validation results, although Model 1 outperformed Model 2 in the 0.3–0.5 probability range, Model 2 outperformed Model 1 in the 0.5–0.8 probability range. Both prediction models slightly underestimated THPT risk at low-risk levels and slightly overestimated it at high-risk levels (Figure 4).

**TABLE 7:** Internal validation using the bootstrap method for the THPT prediction models.

	Model 1	Model 2
ROC AUC obtained through bootstrap resampling	0.91	0.94
Slope (BOC)	0.11	0.16
Mean absolute error	0.03	0.03
Mean squared error	0.00	0.00
0.9 Quantile of absolute error	0.06	0.08

*BOC*, bootstrap optimism corrected*; ROC AUC*, receiver operating characteristic area under the curve.

## Discussion

THPT is a complication often observed after KTx, and post-transplant PTx or calcimimetic induction is often necessary [10, 11]. In this study, including the pre-transplant calcimimetic use and dose information as a predictive factor improved the accuracy of THPT prediction. From the scatter plot of the predicted probabilities of Model 1 and Model 2, the addition of pre-transplant calcimimetic information enhanced the accuracy of prediction of THPT risk in most cases in both the THPT and non-THPT groups, leading to high CNRI values. However, although the ROC AUC of Model 2 was significantly better than that of Model 1, the degree of improvement was relatively modest, contrary to the high CNRI value. In other words, Model 1 was able to predict THPT reasonably well even without pre-transplant calcimimetic information. This is probably because the proportion of patients who had received pre-transplant calcimimetic treatment was not as high, at 25% of the entire cohort. However, the subgroup analysis showed that patients treated with pre-transplant calcimimetics and at higher doses had greatly improved predictive probability. Thus, the larger the proportion of patients receiving pre-transplant calcimimetics and the calcimimetic dose in a cohort, the greater the contribution of calcimimetic information to THPT prediction improvement.

From the kidney graft function and prognosis perspective, pre-transplant PTx may be considered for cases with high THPT risk. For pre-transplant PTx to be properly performed, accurate THPT prediction is indispensable; however, research on THPT prediction models remains limited. Hong et al. [21] developed an excellent predictive model for THPT based on Ca, PTH, and dialysis duration. That study was a pioneering one on THPT prediction and holds significant importance for the prevention and early treatment of THPT. Yet, in that report, there was no mention of a relationship between calcimimetic use and THPT risk. In Japan, since the introduction of cinacalcet in 2008, the number of PTx in dialysis patients has drastically decreased [46]; however, the proportion of post-transplant hyperparathyroidism has not seen a corresponding decrease [3]. Calcimimetics are highly effective against SHPT; however, significant reductions in both PTH and calcium levels may lead to consequent underestimation of THPT risk for patients who should ideally undergo pre-transplant PTx. Therefore, in regions where calcimimetics are widely used, there is a potential risk of misestimating THPT risk.

To the best of our knowledge, this study represents the first report to validate a THPT prediction model that includes pre-transplant use and dose information of calcimimetics. By incorporating pre-transplant calcimimetic information into the predictive model, it becomes possible to properly assign high-THPT risk cases with suppressed PTH and Ca levels under calcimimetic treatment to the high-risk group. This contributes to pre-transplant PTx decision-making without discontinuing calcimimetics. In the context of widespread calcimimetic treatment, information on calcimimetic use and dose would be important for accurate THPT risk prediction.

As THPT prediction advances, candidates for pre-transplant PTx may be identified more frequently. However, the validity of postponing already scheduled KTx for the purpose of pre-transplant PTx remains uncertain. This is because the extension of dialysis duration is associated with poor patient and graft outcomes [47, 48]. The lack of evidence on whether the benefits of pre-transplant PTx outweigh those of shorter dialysis duration is a factor in this uncertainty. Therefore, the timing of PTx should be carefully considered on a case-by-case basis.

This study had some limitations. First, this was a single-center, retrospective study. Second, serum phosphorus data were lacking to evaluate its clinical relevance as a key factor influencing PTH levels [49]. Third, assessment of parathyroid gland size is another challenge as noted in a previous study [50]. There is a certain concern in reproducibility of ultrasound-guided parathyroid gland size measurement. Fourth, the prediction models were not externally validated. Fifth, our cohort was predominantly composed of patients receiving KTx from living donors, a scenario unique to Japan and distinct from Western countries. In addition, the prevalence of calcimimetic use and dialysis practices may differ between countries. Therefore, the prediction models used in this study may not be effective in predicting THPT in KTx candidates from other countries. However, the strengths of this study include the simplicity of the development methods for the prediction models and the use of analytical techniques with free statistical software. Thus, replicating the methods of this study in various cohorts from different regions using patient data would enable the convenient and cost-effective creation of an accurate predictive model.

In conclusion, information on pre-transplant calcimimetic use and dose improved the accuracy of post-KTx THPT prediction. The THPT prediction model that included pre-transplant calcimimetic use and dose information as a predictive factor can contribute to the prevention and early treatment of THPT in the era of calcimimetics. Future studies should perform external validations using new cohorts or cohorts from other institutions.

## Data Availability

The raw data supporting the conclusion of this article will be made available by the authors, without undue reservation.

## Footnotes

1
www.srl-group.co.jp

2
www.tosoh.co.jp
