# Supplementary material for: Pre-Transplant Calcimimetic Use and Dose Information Improves the Accuracy of Prediction of Tertiary Hyperparathyroidism after Kidney Transplantation: A Retrospective Cohort Study
Source: Transpl Int. 2024 May 1;37:12704. doi: 10.3389/ti.2024.12704 (PMC11095396; doi:10.3389/ti.2024.12704)
Supplement: Supplementary file 1 [file Table1.docx]

| **Table S1** Shapiro-Wilk normality test for continuous variables | | |
| --- | --- | --- |
|  | W | *P* value |
| Recipient age (years) | 0.962 | <0.001* |
| Body mass index (kg/m^2^) | 0.996 | 0.164 |
| Parathyroid gland size (mm) | 0.649 | <0.001* |
| Calcimimetic dose per unit of body weight (mg/kg) | 0.448 | <0.001* |
| Corrected calcium before KTx (mg/dL) | 0.991 | 0.002* |
| Intact PTH before KTx (pg/mL) | 0.550 | <0.001* |
| Corrected calcium 1 year post-KTx (mg/dL) | 0.960 | <0.001* |
| Intact PTH 1 year post-KTx (pg/mL) | 0.758 | <0.001* |
| Recipient eGFR 1year post-KTx (mL/min/1.73m^2^) | 0.932 | <0.001* |
| Follow up after KTx (months) | 0.953 | <0.001* |
| *eGFR*, estimated glomerular filtration rate; *KTx*, kidney transplantation; *PTH*, parathyroid hormone. | | |
| **P*-value < 0.05 | | |
