# Supplementary figures and images for: Pre-Transplant Calcimimetic Use and Dose Information Improves the Accuracy of Prediction of Tertiary Hyperparathyroidism after Kidney Transplantation: A Retrospective Cohort Study
Source: Transpl Int. 2024 May 1;37:12704. doi: 10.3389/ti.2024.12704 (PMC11095396; doi:10.3389/ti.2024.12704)

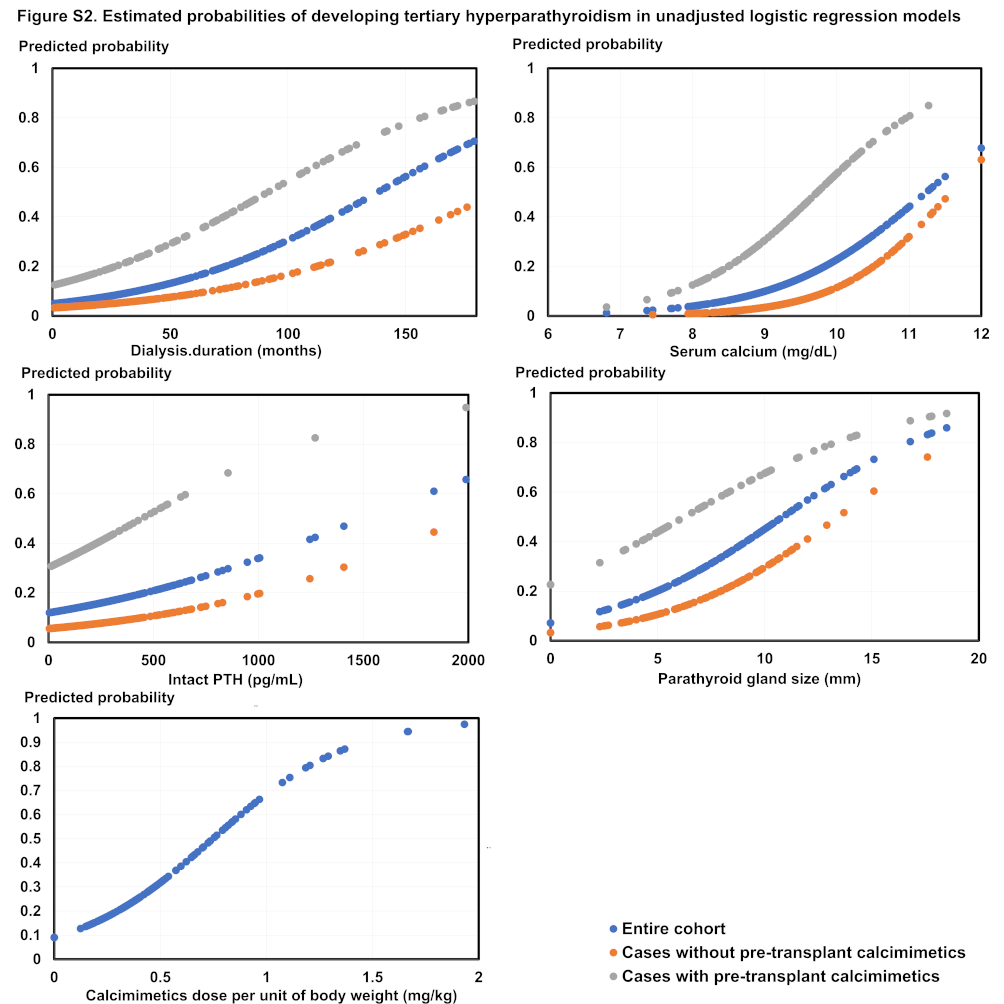

Supplement: Supplementary file 2 [file Image2.tif]

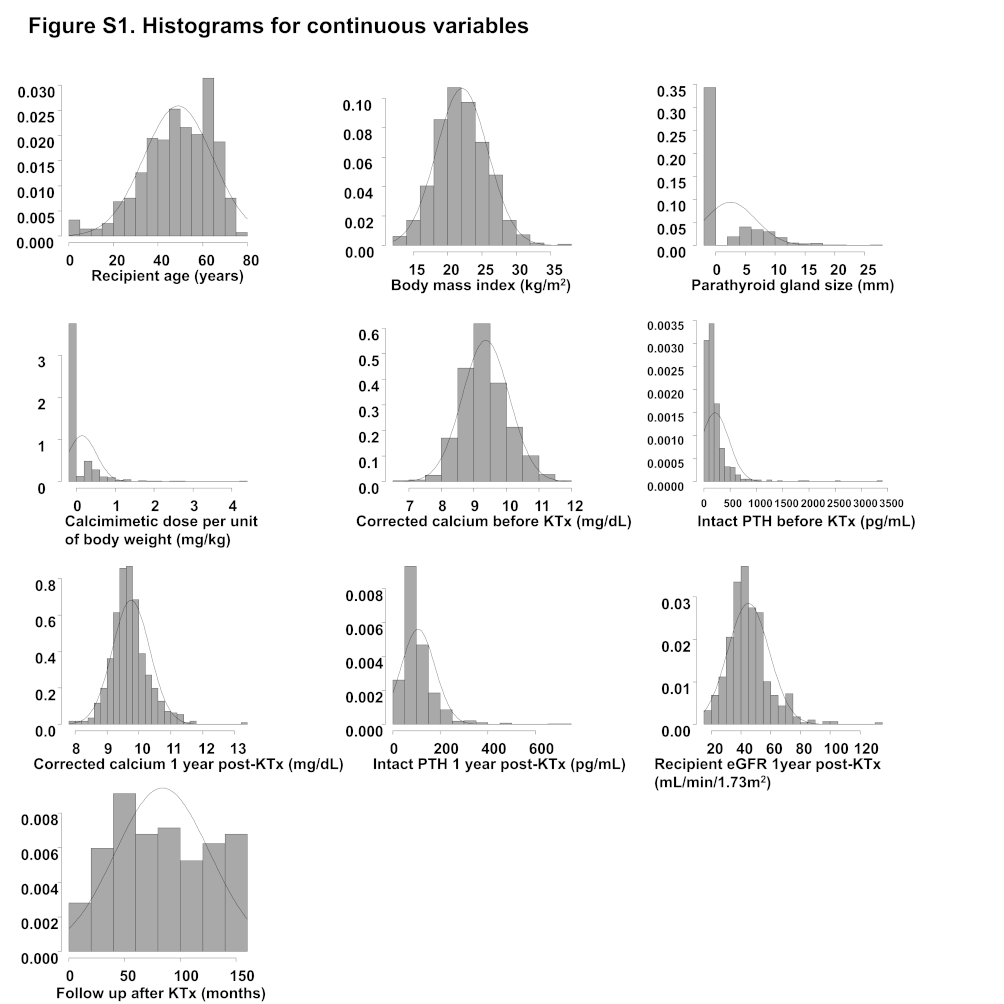

Supplement: Supplementary file 3 [file Image1.tif]
