# Supplementary material for: Pre-Transplant Calcimimetic Use and Dose Information Improves the Accuracy of Prediction of Tertiary Hyperparathyroidism after Kidney Transplantation: A Retrospective Cohort Study
Source: Transpl Int. 2024 May 1;37:12704. doi: 10.3389/ti.2024.12704 (PMC11095396; doi:10.3389/ti.2024.12704)
Supplement: Supplementary file 4 [file Table2.docx]

| **Table S2** Breakdown of pre-transplant calcimimetics dose. | |
| --- | --- |
|  | N = 139 |
| Cincacalcet (mg/day) | 89 |
| 12.5 | 10 |
| 25.0 | 52 |
| 50.0 | 16 |
| >50.0 | 11 |
| Evocalcet (mg/day) | 36 |
| 1.0 | 24 |
| 2.0 | 9 |
| 3.0 | 2 |
| 4.0 | 1 |
| >4.0 | 0 |
| Etelcalcetide (mg/week) | 14 |
| 2.5 | 1 |
| 5.0 | 1 |
| 7.5 | 7 |
| 10.0 | 2 |
| >10.0 | 3 |
