# Supplementary material for: Pre-Transplant Calcimimetic Use and Dose Information Improves the Accuracy of Prediction of Tertiary Hyperparathyroidism after Kidney Transplantation: A Retrospective Cohort Study
Source: Transpl Int. 2024 May 1;37:12704. doi: 10.3389/ti.2024.12704 (PMC11095396; doi:10.3389/ti.2024.12704)
Supplement: Supplementary file 5 [file Table3.docx]

| **Table S3** Patients characteristics classified by presence or absence of pre-transplant calcimimetic treatment | | | |
| --- | --- | --- | --- |
|  | Patients without  pre-transplant calcimimetic treatment  N = 415 | Patients with  pre-transplant calcimimetic treatment  N = 139 | *P*-value |
| Recipient age (years, IQR) | 50 (39–62) | 52 (38–61) | 0.888 |
| Recipient sex (male, %) | 262 (61.0) | 90 (64.7) | 0.810 |
| Body mass index (kg/m^2^, SD) | 22.0 (3.8) | 22.3 (3.6) | 0.442 |
| Dialysis duration (months, IQR) | 16 (5–41) | 45 (15–110) | <0.001* |
| Living donor (%) | 389 (93.7) | 117 (84.2) | 0.001* |
| Original disease (%) |  |  | 0.308 |
| Glomerular disease | 141 (34.0) | 51 (36.7) |  |
| Diabetic kidney disease | 113 (27.2) | 28 (20.1) |  |
| Polycystic kidney disease | 20 (4.8) | 8 (5.8) |  |
| Hypertensive kidney disease | 32 (7.7) | 6 (4.3) |  |
| Others | 35 (8.4) | 14 (10.1) |  |
| Unknown | 74 (17.8) | 32 (23.0) |  |
| Preformed DSA (%) | 36 (8.7) | 4 (2.9) | 0.036* |
| ABO blood type incompatible kidney transplantation (%) | 127 (30.6) | 33 (23.7) | 0.151 |
| Parathyroid gland size (mm, IQR) | 7.0 (4.9–9.3) | 8.0 (5.3–10.3) | 0.050 |
| VDRA before KTx | 253 (61.0) | 99 (71.2) | 0.038* |
| Lab data before KTx |  |  |  |
| Corrected calcium (mg/dL, IQR) | 9.3 (9.0–9.8) | 9.4 (8.8–9.9) | 0.417 |
| Intact PTH (pg/mL, IQR) | 154.0 (89.0–250.0) | 175 (77.4–247.5) | 0.643 |
| Recipient eGFR (mL/min/1.73m^2^, IQR) | 4.8 (3.8–6.1) | 3.9 (3.4–5.5) | <0.001* |
| *DSA*, donor-specific HLA antibody; *eGFR*, estimated glomerular filtration rate; *IQR*, interquartile range; *KTx*, kidney transplantation; *PTH*, parathyroid hormone; *SD*, standard deviation; *VDRA*, vitamin D receptor activator. | | | |
| **P*-value < 0.05 | | | |
| The results of parathyroid gland size excluded patients in whom parathyroid gland was not detected by echography. | | | |
