# Supplementary material for: Pre-Transplant Calcimimetic Use and Dose Information Improves the Accuracy of Prediction of Tertiary Hyperparathyroidism after Kidney Transplantation: A Retrospective Cohort Study
Source: Transpl Int. 2024 May 1;37:12704. doi: 10.3389/ti.2024.12704 (PMC11095396; doi:10.3389/ti.2024.12704)
Supplement: Supplementary file 6 [file Table4.docx]

| **Table S4** DeLong's test for two correlated ROC curves (output of the calculation by EZR) | |
| --- | --- |
| data: Predicted probability (Model1) and Predicted probability (Model 2) in Dataset by THPT (0, 1) | |
| Z = -3.4694, *p*-value = 0.0005216 | |
| alternative hypothesis: true difference in AUC is not equal to 0 | |
| 95 percent confidence interval: -0.04217671 -0.01172568 | |
| sample estimates: | |
| AUC of roc1 | AUC of roc2 |
| 0.9253612 | 0.9523124 |
